# Regional citrate anticoagulation for intermittent hemodialysis in the intensive care: what is the optimal setup?

**DOI:** 10.1186/s13613-021-00858-w

**Published:** 2021-05-03

**Authors:** Jakob Gubensek, Vanja Persic

**Affiliations:** 1 Center for Acute and Complicated Dialysis, Department of Nephrology, University Medical Center Ljubljana, Zaloska 2, 1000 Ljubljana, Slovenia; 2 Faculty of Medicine, University of Ljubljana, Vrazov trg 2, Ljubljana, Slovenia

Leroy et al. recently published a propensity score‑matched cohort study comparing regional citrate anticoagulation (RCA) and heparin anticoagulation during intermittent hemodialysis in intensive care unit (ICU) patients [1]. The study reports their experience in introducing RCA for intermittent hemodialysis in the ICU setting. The procedures were performed by intensivists with regular dedicated training. They observed comparable dialysis efficacy but a significantly higher number of circuit clotting (12.9% vs. 2.4%, *p* = 0.02) and interruptions due to high transmembrane pressure (21% vs. 7%, *p* = 0.02), resulting in premature termination of > 30% of RCA sessions [1]. In adjusted propensity analysis, RCA was also associated with an increased risk of circuit clotting (absolute difference = 0.10, 95% CI [0.03–0.18], *p* = 0.008) [1]. This observation is in contrast to what has been observed in several studies with continuous methods (CRRT). Better circuit life-time in RCA-CRRT is also the reason, why RCA is recommended as the first-line anticoagulation method for CRRT in the KDOQI guidelines.

In general, there are two setups for RCA, depending on how calcium is substituted. While protocols initially used a calcium-free dialysate and a separate calcium infusion, later protocols were developed with a calcium-containing dialysate, claiming simplicity and safety. Simplicity results from using a “regular”, calcium-containing dialysate and omission of routine calcium infusion. Better safety results from reduced likelihood of inadvertent severe hypocalcemia. Both are questionable, as additional calcium by separate infusion is necessary in 3.4% [2] to 8% [1] of sessions with calcium-containing dialysate, therefore, monitoring of ionized calcium remains mandatory. Even with the simplicity and safety of procedure performed by intensivists in mind, the use of calcium-containing dialysate is, therefore, questionable.

In their study [1], the authors used RCA protocol with calcium-containing dialysate and adequately discuss clotting problems in light of commonly used 1.5 mmol/L calcium dialysate. We have shown very nicely in a small randomized study in chronic hemodialysis that, as compared to calcium-free dialysate, even a 1.25 mmol/L calcium dialysate was associated with an unacceptably common venous line exchange due to venous bubble-trap clotting (24%) and premature procedure termination (16%) [3], results similar to the present study [1] and to what is reported for heparin-free dialysis. As we have shown, this results from insufficiently low ionized calcium in the venous part of circuit (0.63 ± 0.11 mmol/L), as a result of calcium-influx from dialysate, despite optimal pre-dialyzer values (0.24 ± 0.05 mmol/L) [3]. When calcium-containing dialysate is used, calcium should be low (1.25 mmol/l) and the citrate dose should be increased (approx. 4 mmol/l of blood); with this setup > 97% success rate was reported [2]. However, the optimal way that ensures excellent anticoagulation throughout the circuit probably includes a sufficient dose of citrate (approx. 3 mmol/L of blood, equivalent to approx. < 0.30 mmol/L ionized calcium), providing anticoagulation up to the middle of the dialyzer, where most of citrate is removed, and a calcium-free dialysate, which enables persistence of severe hypocalcemia and efficient anticoagulation in the venous part of the circuit [3, 4]. Our experience with such setup also in ICU setting suggests both an excellent safety profile along with a high efficiency; our experience is not published, but excellent safety and efficacy results were published by Apsner, although in chronic hemodialysis population [4].

Recently, an innovative method for performing regional anticoagulation has been described in which anticoagulation is achieved by lowering ionized calcium using a calcium-free dialysate containing a low concentration of citrate without a separate citrate infusion [5]. As we have already discussed in our comment on the original article, although a low concentration of citrate in the dialysate might contribute slightly to the anticoagulant effect in the venous part of the circuit, we believe that it is the diffusion of calcium against the calcium-free dialysate which mostly contributes to severe post-filter hypocalcemia. Therefore, this new protocol also highlights the importance of using calcium-free dialysate for efficient anticoagulation.

Regarding the safety of using calcium-free dialysate, it should be emphasized that although data from chronic hemodialysis show very good safety [4], this remains to be confirmed in the more vulnerable ICU population. Although some authors claim that calcium monitoring is not necessary in some settings [5], we consider it mandatory to exclude human error in preparing calcium infusion or mixing arterial and venous lines, which rarely does occur and can lead to severe hypocalcemia with potentially fatal consequences.

To conclude, the use of calcium-containing dialysate with RCA often results in unacceptably high rates of circuit failure. To achieve excellent biocompatibility and circuit patency commonly and rightly attributed to RCA, it should be performed in an optimal setup which, in our opinion, includes a citrate infusion and a calcium-free dialysate, providing anticoagulation throughout the circuit. This setup is common in maintenance dialysis and we propose its efficacy and safety is tested also in ICU setting.

## Data Availability

Not applicable.

## Abbreviations

CRRT: Continuous renal replacement therapy
ICU: Intensive Care Unit
RCA: Regional Citrate Anticoagulation

## References

[1] Leroy C, Pereira B, Soum E, Bachelier C, Coupez E, Calvet L (2021). Comparison between regional citrate anticoagulation and heparin for intermittent hemodialysis in ICU patients: a propensity score-matched cohort study. Ann Intensive Care.

[2] Fiaccadori E, Regolisti G, Cademartiri C, Cabassi A, Picetti E, Barbagallo M (2013). Efficacy and safety of a citrate-based protocol for sustained lowefficiency dialysis in AKI using standard dialysis equipment. Clin J Am Soc Nephrol.

[3] Buturovic-Ponikvar J, Cerne S, Gubensek J, Ponikvar R (2008). Regional citrate anticoagulation for hemodialysis: calcium-free vs. calcium containing dialysate—a randomized trial. Int J Artif Organs..

[4] Apsner R, Buchmayer H, Gruber D, Sunder-Plassmann G (2005). Citrate for long-term hemodialysis: prospective study of 1,009 consecutive high-flux treatments in 59 patients. Am J Kidney Dis.

[5] Faguer S, Saint-Cricq M, Nogier M-B, Labadens I, Lavayssiere L, Kamar N (2017). Heparin-free prolonged intermittent hemodialysis using calcium-free citrate dialysate in critically ill patients. Crit Care Med.

